# Defining an Ultra-Low Risk Group in Asymptomatic IgM Monoclonal Gammopathy

**DOI:** 10.3390/cancers13092055

**Published:** 2021-04-23

**Authors:** David F. Moreno, Arturo Pereira, Natalia Tovar, María Teresa Cibeira, Laura Magnano, María Rozman, Mónica López-Guerra, Dolors Colomer, Beatriz Martín-Antonio, Raquel Jiménez-Segura, Ignacio Isola, Luis Gerardo Rodríguez-Lobato, Aina Oliver-Caldés, Mari Pau Mena, Laura Rosiñol, Joan Bladé, Carlos Fernández de Larrea

**Affiliations:** 1 Amyloidosis and Myeloma Unit, Department of Hematology, Hospital Clínic of Barcelona, 08036 Barcelona, Spain; dfmoreno@clinic.cat (D.F.M.); ntovar@clinic.cat (N.T.); mcibeira@clinic.cat (M.T.C.); beatriz.antonio@quironsalud.es (B.M.-A.); rjimene1@clinic.cat (R.J.-S.); imisola@sjdhospitalbarcelona.org (I.I.); lgrodriguez@clinic.cat (L.G.R.-L.); oliver@clinic.cat (A.O.-C.); mmenaj@clinic.cat (M.P.M.); lrosinol@clinic.cat (L.R.); jblade@clinic.cat (J.B.); 2 Institut d’Investigacions Biomèdiques August Pi i Sunyer (IDIBAPS), 08036 Barcelona, Spain; apereira@clinic.cat (A.P.); lcmagnan@clinic.cat (L.M.); mrozman@clinic.cat (M.R.); lopez5@clinic.cat (M.L.-G.); dcolomer@clinic.cat (D.C.); 3 Department of Hemotherapy and Hemostasis, Hospital Clínic of Barcelona, 08036 Barcelona, Spain; 4 Hematopathology Unit, Department of Pathology, Hospital Clínic of Barcelona, 08036 Barcelona, Spain; 5 Centro de Investigación Biomédica en Red-Oncología (CIBERONC), 28029 Madrid, Spain

**Keywords:** IgM MGUS, smoldering Waldenström macroglobulinemia, immunoparesis, bone marrow

## Abstract

**Simple Summary:**

Patients with asymptomatic IgM monoclonal gammopathies include IgM monoclonal gammopathy of undetermined significance (IgM MGUS) and smoldering Waldenström macroglobulinemia (SWM), all with some risk of progression to symptomatic Waldenström macroglobulinemia, amyloidosis, or other lymphoproliferative disorder. Due to their low incidence, few studies have focused on the risk of progression, with SWM being the most studied. As both are recognized clinical-pathological entities that share similar clonal and phenotypical features, we focus on defining new biomarkers of progression in this population with long follow-up.

**Abstract:**

We analyzed 171 patients with asymptomatic IgM monoclonal gammopathies (64 with IgM monoclonal gammopathy of undetermined significance—MGUS and 107 with smoldering Waldenström macroglobulinemia - SWM) who had a bone marrow (BM) evaluation performed at diagnosis. Abnormal free-light chain ratio (53% vs. 31%) and *MYD88* mutation prevalence (66% vs. 30%) were higher in patients with SWM. No other differences were found among groups. With a median follow-up of 4.3 years, 14 patients progressed to Waldenström macroglobulinemia, 1 to amyloidosis, and 28 died without progression. The *MYD88* mutation was found in 53% of patients (available in 160 patients). Multivariate analysis showed that immunoparesis (subhazard ratio—SHR 10.2, 95% confidence interval—CI: 4.2–24.8; *p* < 0.001) and BM lymphoplasmacytic infiltration ≥ 20% (SHR: 6, 95% CI: 1.6–22.1; *p* = 0.007) were associated with higher risk of progression. We developed a risk model based on these two risk factors. In the absence of both variables, an ultra-low risk group was identified (SHR 0.1, 95% CI 0.02–0.5; *p* = 0.004), with 3% and 6% of cumulative incidence of progression at 10 and 20 years, respectively. Bootstrap analysis confirmed the reproducibility of these results. This study finds immunoparesis and BM infiltration as biomarkers of progression as well as a low-risk group of progression in asymptomatic IgM monoclonal gammopathies.

## 1. Introduction

Waldenström macroglobulinemia (WM) is a lymphoproliferative disorder characterized by the presence of an IgM monoclonal protein (M-protein) and bone marrow (BM) lymphoplasmacytic infiltration [1,2]. It is preceded by two asymptomatic clinicopathological entities such as IgM monoclonal gammopathy of undetermined significance (MGUS) and smoldering WM (SWM) [2,3,4].

IgM MGUS predominates in the elderly, so most patients may live their remaining lifespan without any sign of progression to WM or other malignant disorder [5,6]. On the other hand, SWM has a clear increased risk of progression but varies between studies [3,4,7]. Regarding the risk of progression, it has been described that the M-protein size, free light chain (FLC) ratio, serum albumin level, and reduction of one or two uninvolved immunoglobulin isotype levels (immunoparesis) as predictors of progression from IgM MGUS [5,8,9]. Moreover, SWM shares some of the risk factors above mentioned, with the addition of BM tumor load and β2-microglobulin [4]. However, there are two definitions of SWM according to BM disease. The Mayo Clinic criteria established a cut-off of 10% while the Second International Consensus on Waldenström macroglobulinemia defined SWM as any BM lymphoplasmacytic infiltration in the absence of symptoms [1,2,3]. So far, risk models have been developed under these definitions applied to each clinical entity among centers. Only one study proposed the inclusion of IgM MGUS and SWM in a unique and feasible risk model as both entities share some prognostic determinants but it has not been replicated [7].

As a result of this, the reproducibility of all these prognostic factors across studies is low, at least in part because of the low incidence of the disease and their protracted natural history. The aim of this study was to investigate predictors of progression in patients with asymptomatic IgM monoclonal gammopathies observed over a long period, incorporating immunoparesis, BM infiltration, and the presence of *MYD88* L265P mutation with an intention to find an accessible and reproducible risk model overtaking the definition gap and highlighting a population of patients that may be categorized as IgM MGUS or SWM. 

## 2. Materials and Methods

### 2.1. Patient Characteristics

Medical records of 206 patients with asymptomatic IgM monoclonal gammopathies diagnosed in our institution from 1982 to 2018 were reviewed. Bone marrow aspirate was available in 171 patients, which was our final study population. The Ethics Committee of the Hospital Clinic of Barcelona provided institutional review board approval for this study.

IgM MGUS and SWM diagnoses were included in the present study and defined by the Mayo Clinic criteria [1,3]. At the time of diagnosis, there was no evidence of target organ involvement according to international consensus, such as constitutional symptoms, anemia, hyperviscosity, enlarged lymph nodes, or peripheral neuropathy [10,11]. 

Variables were grouped into categorical (sex, immunoparesis, abnormal FLC ratio, *MYD88* L265P mutation, M-protein size, IgM serum concentration, serum albumin, and β2-microglobulin levels), continuous (age, calcium, creatinine, hemoglobin), and time-to-event (progression, death). Immunoparesis was defined as a decreased concentration in both uninvolved serum immunoglobulin isotypes below the normal range (IgG < 6.8 g/L, IgA < 0.66 g/L). Standard nephelometry was used to measure immunoglobulins. A normal serum FLC ratio (The Binding Site Group Ltd., Birmingham, UK) was defined as 0.26 to 1.65 according to multiple myeloma guidelines.

### 2.2. Bone Marrow Evaluation

Morphology was reviewed after May-Grunwald-Giemsa staining as stated by standard procedures. A senior cytologist analyzed systematically 200 bone marrow total nucleated cells in two slides from random areas, and the percentages of lymphoplasmacytic and plasma cell infiltration were estimated. Flow cytometry results were not included in this study because of the heterogenous availability of these results over the last 30 years.

### 2.3. MYD88 L265P Mutation Analysis

DNA samples were obtained from bone marrow mononuclear cells and kept at −80 °C. A commercial kit was performed for DNA isolation (DNeasy Blood and Tissue Kit, Qiagen, Germantown, MD, USA). BM samples for molecular biology were available in 160 patients. A conventional allele-specific polymerase chain reaction (PCR) was used to evaluate *MYD88* L265P mutations as previously described [12].

### 2.4. Statistical Analysis

The primary study endpoint was progression to symptomatic WM. A secondary endpoint was survival from the diagnosis of asymptomatic IgM monoclonal gammopathy.

Variables investigated for association with time to progression were selected based on their prognostic relevance in previous studies and clinical meaningfulness. They included patient sex, immunoparesis, IgM concentration (≥45 g/L vs. <45 g/L), abnormal FLC ratio, Bence-Jones proteinuria, β2-microglobulin ≥4.5 mg/dL, serum albumin (≤35 g/L vs. >35 g/L), lymphoplasmacytic infiltration ≥20% in the bone marrow aspirate, and *MYD88* mutation. In previous studies the size of the M-protein had been investigated at several cut-off values, hence, in the present analysis, we dichotomized this variable at the median value in our series (≥12 g/L vs. <12 g/L). 

Survival was estimated by the Kaplan–Meier method and factors predicting mortality were investigated by Cox multivariate regression without previous selection by univariable analysis. All the variables met the proportional hazards assumption as tested by the Grambsch-Therneau test [13]. Cumulative incidence was used to estimate the risk of progression to symptomatic WM in the context of death without progression as a competing risk. Multivariate analysis of factors predicting progression was performed by the method of Fine and Gray [14]. In this regression method, the subdistribution hazard ratios (SHR) are equivalent to the HRs in the Cox model. All multivariate models were analyzed by parsimonious stepwise backward elimination and reconsideration of variables, based on association with the endpoint and clinical meaning. Internal validity of models predicting disease progression was evaluated by bootstrapping [15]. In brief, 1000 samples the size of the whole series were taken randomly with reposition so that, in each sample, individual patients may be represented once, more than once, or not represented at all. Prognostic models were then estimated in the 1000 samples and the proportion of samples yielding a significant association with progression was recorded. All the analysis was performed with SPSS version 25 (IBM Corp, Armonk, NY, USA) and Stata version 11 (StataCorp, College Station, TX, USA).

## 3. Results

### 3.1. Baseline Characteristics

The median age of the 171 patients at the diagnosis of IgM MGUS or SWM was 69 years (range: 28–98), and 90 (53%) were males. There were 10 patients younger than 50 years (6%) and only one younger than 30. Table 1 summarizes the main laboratory results at the time of diagnosis. The M-protein size spread over a wide range, from unmeasurable (only detectable by immunofixation but not by conventional serum electrophoresis) to 28.2 g/L. The M-protein size was >12 g/L in 53% of patients, and >15 g/L in 23%. 29% of the patients were diagnosed before 2008, and 71% from 2008 to 2018.

According to the Mayo Clinic diagnostic criteria, we found 64 (37%) patients with IgM MGUS and 107 (63%) patients with SWM. Baseline characteristics were similar between potential risk factors, only showing differences in the BM infiltration rate (5% vs. 23%; *p* < 0.001), abnormal serum FLC ratio (31% vs. 53%; *p* = 0.04), and the *MYD88* mutation detection (30% vs. 66%; *p* < 0.001) as shown in Table 2. 

### 3.2. Outcomes and Risk Factors

After a median follow-up of 4.3 years (IQR: 2.3–7.7), 14 patients had progressed to symptomatic WM, 1 to amyloidosis, and 28 had died without progression. The initiation of treatment of each patient who progressed was mainly due to anemia (nine patients), followed by symptomatic lymphadenopathy (three patients) and peripheral neuropathy with progressive increase of the M-protein size (two patients). None of the patients progressed to IgM-related disorder without any other sign of progression to WM. Progressive disease was documented at least after one year of close follow-up and confirmed with pathology and imaging studies. Regarding each diagnosis, progressive disease was present in 5% of patients with IgM MGUS and 10% of patients with SWM.

**Figure 1:**
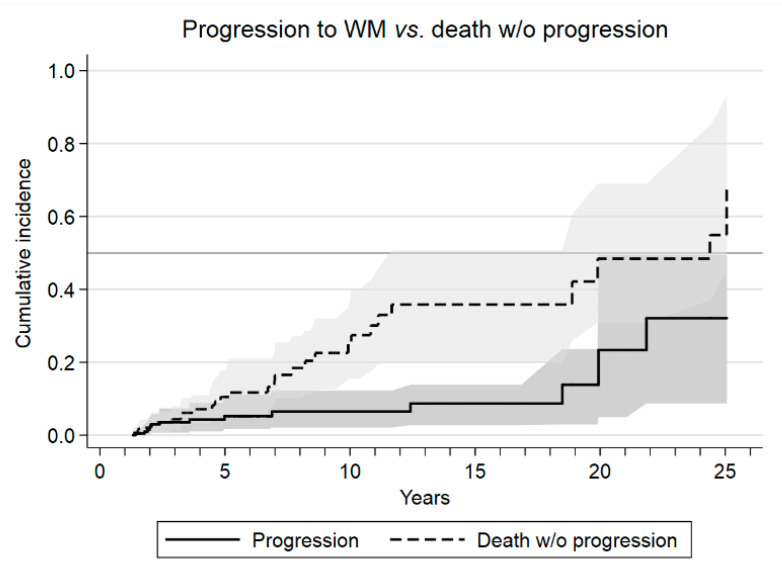
Relative risk of progression to Waldenström macroglobulinemia (WM) or death without (*w*/*o*) progression evaluated as cumulative incidence in the framework of competing events.

Eleven patients were lost to follow-up after a median of 2.6 years (IQR: 2.0–4.0) and were censored at the time of the last contact. The projected median overall survival (OS) was 17.2 years (95% CI: 10.8–28.4), and 38% of patients are expected to survive more than 20 years (Supplementary Figure S1). In the competing risks analysis, the cumulative incidence of progression at 5, 10, and 15 years was 5.2%, 6.5%, and 8.7%, respectively, whereas the equivalent values for death without progression were 10.5%, 27.5%, and 36.0%, respectively (Figure 1). Considering diagnosis, the cumulative incidence of progression at 5 and 10 years from diagnosis were 4% in IgM MGUS compared to 8% and 12% in SWM patients, respectively. 

**Figure 2:**
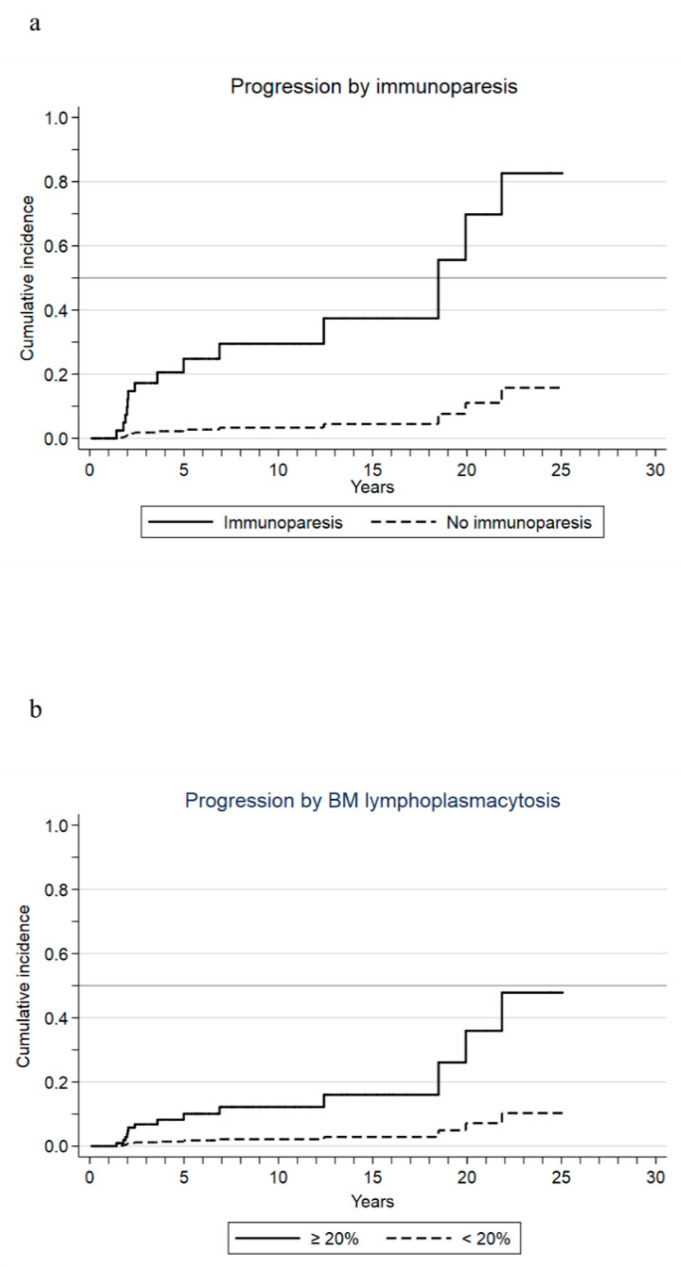
Cumulative incidence of progression in the framework of death as a competing event, according to immunoparesis (**a**) and bone marrow (BM) lymphoplasmacytic infiltration (**b**) at diagnosis of asymptomatic IgM monoclonal gammopathy.

**Figure 3:**
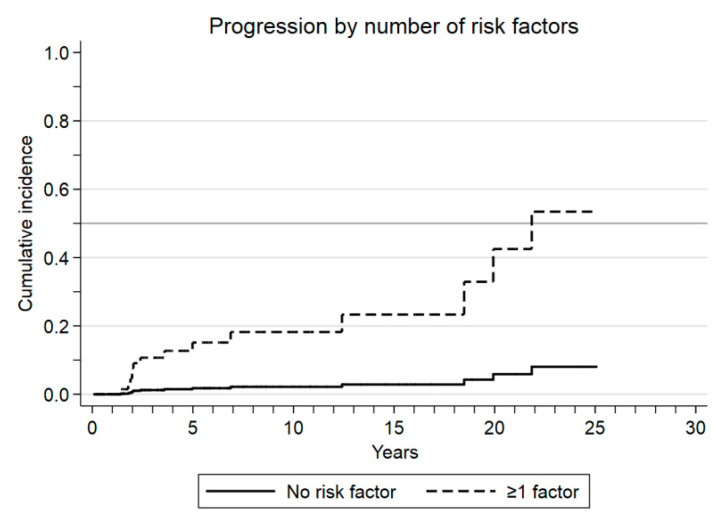
Cumulative incidence of progression in the framework of death as a competing event according to the number of risk factors at the diagnosis of asymptomatic IgM monoclonal gammopathy (immunoparesis and bone marrow lymphoplasmacytosis ≥20%).

Parsimonious multivariate analysis of factors associated with progression to WM in the context of death as a competing risk identified immunoparesis (SHR: 10.2, 95% CI: 4.2–24.8; *p* < 0.001) and lymphoplasmacytic infiltration ≥ 20% in the BM (SHR: 6, 95% CI: 1.6–22.1; *p* = 0.007) as the only statistically significant predictors of progression (Figure 2a,b). Out of the 171 patients, 167 were introduced in the risk model. One hundred patients did not have any risk factor, 67 at least one, and 8 patients had both at diagnosis. Our risk model concluded that the absence of both immunoparesis and BM lymphoplasmacytosis at diagnosis predicted a very low risk of progression to symptomatic WM as compared with the presence of at least one risk factor (SHR: 0.1, 95% CI 0.02–0.5; *p* = 0.004; Table 3). Cumulative incidence of progression for patients who had none of these risk factors was 3% and 6% at 10 and 20 years, respectively, as compared with 19% and 42% for those with at least one risk factor (Figure 3). The absence of both factors was confirmed as a statistically significant predictor of low risk of progression in 100% of 1000 bootstrap samples giving optimal internal validity not dependent on the specific composition of our series.

BM lymphoplasmacytic infiltration ≥20% was associated with shorter survival (11.7 vs. 19.9 years; *p* = 0.04), whereas immunoparesis showed no significant statistical association with this outcome (13.0 vs. 18.9 years; *p* = 0.36). With an *MYD88* mutation prevalence of 52.5% in the whole series, we found a trend for this mutation to be associated with worse OS (21.8 years vs. not reached; *p* = 0.06) and a lower prevalence in patients with less than 20% of lymphoplasmacytic infiltration (38% vs. 77%; *p* < 0.001). 

Due to the relevant prognostic significance of immunoparesis in our series, we investigated the association between this biomarker and risk factors for disease progression previously described in IgM MGUS and SWM. As summarized in Supplementary Table S1, immunoparesis was significantly associated with a larger M-protein size, higher lymphoplasmacytic infiltration in the BM, and abnormal FLC ratio. We also found a trend towards lower serum albumin levels in patients with immunoparesis. 

## 4. Discussion

In the present study, immunoparesis was identified as a strong predictor of progression from asymptomatic IgM monoclonal gammopathy to symptomatic WM. Indeed, absence of immunoparesis together with a BM lymphoplasmacytic infiltration <20% defined a particularly good prognosis population with only a 3% cumulative incidence of progression at 10 years and 6% at 20 years, with a 90% reduction in the progression risk compared to patients with at least one of them. To the best of our knowledge, this is the first report on the prognostic significance of immunoparesis in this group of patients with long follow-ups. Furthermore, our risk model may overtake the diagnostic bias, fitting patients with IgM MGUS or SWM whether using the Mayo Clinic or the Second International Consensus diagnostic criteria.

Our study population was uniform regarding initial clinical characteristics as shown in Table 2. Diagnosis was made on the findings of BM aspirate by expert hematopathologists and progressive disease was confirmed by BM biopsy and imaging studies. The BM aspirate is a feasible and reliable tool to diagnose IgM monoclonal gammopathies. In this sense, there are studies from other centers where their diagnostic criteria or their risk models relied on the basis of bone marrow aspirates [3,7,16,17]. While most of our patients were diagnosed in the last two decades of the study; progressive disease was observed at similar rates across the decades.

The initiation of treatment of each patient who progressed was similar to that described by the International Consensus [18,19]. All patients progressed after a close follow-up of at least one year, so no patient could have been considered as early slow-growing symptomatic WM. We highlight that the cumulative incidence of progression in our series is lower, being more comparable to a study reported by Alexanian et al. [20] and closer to a low or intermediate group in the external validation series by Bustoros et al. [4]. In the case of IgM MGUS, our series kept a 4% cumulative incidence of progression at 5 and 10 years, comparable to other series [9].

Regarding risk factors, several have been proposed for IgM MGUS and shared by patients with SWM, such as M-protein size, FLC ratio, serum albumin level, and BM infiltration, among others [3,4,5,7,8]. All of them parallel the tumoral load and the immune deregulation. As a result, newly prognostic factors with an indirect measure of disease are emerging. Although not all patients with IgM MGUS or SWM harbor the *MYD88* mutation, it is a promising marker already used in risk models of progression [21]. The CXCL13 levels also seemed to be a reliable biomarker that resembles BM tumoral load [22]. Another promising biomarker is immunoparesis, which has previously been reported as a predictor for disease progression in patients with IgM MGUS but not replicated by others [5,8,21]. In the case of SWM, it was also reported as a potential risk factor of progression to symptomatic WM [3,23]; however, it was not included in the study by Bustoros et al. [4]. 

It is worth noting that, in our study, patients with immunoparesis had significantly larger M-protein size, more frequent abnormal FLC ratios, and a trend to lower serum albumin levels. It can be hypothesized, therefore, that all these prognostic biomarkers emerge from the same pathological process resulting in immunoparesis, and that identification of one or another marker as a statistically significant predictor might be contingent on the composition of each patient series. In this regard, a first step to validate the prediction model in our series was done by bootstrapping internal validation. It confirmed that the good prognosis group from our study (no immunoparesis and less than 20% of BM infiltration) is able to perform well in our center. As the next step implies the use of external series, we consider that our parsimonious prediction model could be easily implemented by other groups in order to confirm and validate these results according to clinical and laboratory practices in other centers.

Other factors may have contributed to explaining the discordance between our results and those previously published on the prognosis of IgM MGUS and SWM. In our series, nearly half the patients lacked information on the FLC ratio at diagnosis because they were first seen before the test was available. This may have reduced the statistical power to detect any influence of this biomarker on the progression rate. Regarding M-protein size, it is the most reproducible predictor of disease progression in patients with IgM MGUS and SWM, and several cut-off values have been put forward as prognostic factors [4,5,7,21,24]. Since the risk of progression parallels the increasing of the M-protein size, choosing one or another cut-off certainly implies some arbitrariness. In our case, we choose the series’ median value (12 g/L), which is a relatively low cut-off because of our initial goal of better defining patients with a low risk of progression. 

Regarding BM infiltration, there is data that supports its value as a prognostic marker of progression in patients with SWM [3,4]. However, it is overlooked in the case of IgM MGUS as it is usually diagnosed based only on serological criteria and the absence of symptoms. The Mayo Clinic series reported that only 12% of patients with MGUS underwent BM evaluation [5]. The Swedish group also reported that 20% of their study group had a BM assessment [9]. According to all diagnostic criteria used to classify patients either with IgM MGUS or SWM, the value of the BM infiltration as a risk factor may vary. To surpass this problem and with an intention to identify common biomarkers, we included all patients that underwent a BM evaluation so we could investigate whether BM infiltration could complement immunoparesis as predictors for disease progression. We established a 20% cut-off value on the basis of previous studies with SWM [3] and smoldering multiple myeloma [25,26].

The combination of both variables, immunoparesis and lymphoplasmacytic infiltration ≥20%, allowed us to identify a group of patients who lacked both biomarkers and had a particularly good prognosis, with cumulative incidences of progression of 3% and 6% at 10 and 20 years from diagnosis of asymptomatic IgM monoclonal gammopathy, showing a very slow and indolent behavior. 

Previous studies have associated a higher incidence of the mutated *MYD88* gene and increased allele burden with a transition from IgM MGUS to SWM and symptomatic WM, and increased lymphoplasmacytic infiltration in the BM [21,27,28,29]. We found the *MYD88* mutation in half the patients who were tested (30% in IgM MGUS and 66% in SWM). This prevalence varies among different studies, techniques, and the diagnostic criteria used. Using allele-specific PCR, our results are less than expected by other reports, especially in patients with SWM. It may be explained by the fact that our global series had less bone marrow infiltration compared to the series by Bustoros et al. (84% MYD88 mutation detection) [4] as well as technical issues using DNA extracted from fixed tissues.

Regarding prognosis, we did not find that patients bearing the mutated gene progressed to WM differently from those bearing the wild gene. Interestingly, the *MYD88* mutation was less prevalent in patients with BM lymphoplasmacytic infiltration <20% and had no association with immunoparesis.

Our study has several strengths and weaknesses. Among the latter, the retrospective design and the long timespan led to incomplete data in some cases, mainly a lack of current biomarkers in patients who were first seen long ago. This also led to less *MYD88* mutation prevalence in patients with SWM; technical issues related to samples may have arisen. It is worth noting that the loss to follow-up rate was kept low despite the old age of patients and the long follow-up. Moreover, while the number of patients from our series is 171, it is not the exception from the reported by other groups. The Mayo Clinic described 210 patients, which is the study with the greatest number of patients with IgM MGUS [5]. It is followed by 118 patients from the Swedish group [9]. Regarding SWM, we included 104 patients in our risk model, which is comparable to the DFCI external validation series [4].

One of the strengths of the present study relies on the use of a competing risks framework to estimate the rates of progression. Many more patients died from causes unrelated to the IgM monoclonal gammopathy than progressed to WM, so the standard Kaplan-Meir analysis would have overestimated the progression rate [30]. Another strength is the high internal validity for this extremely low-risk group. This definition can have clinical significance, as this group has only 6% of cumulative incidence of progression at 20 years. 

## 5. Conclusions

In summary, we identified immunoparesis as an important predictor of progression in asymptomatic IgM monoclonal gammopathy and defined a population of particularly good prognosis based on the absence of this biomarker and a small (<20%) lymphoplasmacytic infiltrate as evaluated by BM aspirate. These findings can help in reassuring good prognosis for these patients and to schedule accordingly the follow-up medical visits.

## Figures and Tables

**Table 1:** Laboratory values in 171 patients with asymptomatic IgM monoclonal gammopathy.

	Median (IQR ^a^)	Frequencies
Serum M-protein (g/L)	12.2 (9.1–14.7)	≥12 in 53.2% ≥15 in 23.4%
Serum IgM (g/L)	5.4 (3.5–11)	<30 in 97%
Calcium (mg/dL)	9.5 (9.2–9.8)	>10.5 in 3.2%
Creatinine (mg/dL)	0.9 (0.8–1)	>2 in 0.6%
Hemoglobin (g/dL)	13.4 (12.2–14.6)	<12 in 15.8%
Platelets (×10^9^/L)	237 (194–291)	<100 in 1.2%
β2-microglobulin (mg/dL)	2.3 (1.9–3.1)	≥4 in 7%
Albumin (g/L)	43 (40–45)	≤35 in 4.1%
Lymphoplasmacytic infiltration	16 (11–24)	
Immunoparesis ^b^ (%)		14/167 (8.4)
Abnormal serum FLC ratio ^c^ (%)		42/92 (45.7)
*MYD88* L265P mutation ^d^ (%)		84/160 (52.5)
Progressive disease (%)		14/171 (8.2)

^a^ IQR: interquartile range; ^b^ immunoparesis defined as a reduction in both uninvolved serum heavy chain immunoglobulin (IgG and IgA); ^c^ FLC: free light chain; ^d^ MYD88 mutation: available in 160 patients.

**Table 2:** Laboratory values in patients categorized by diagnosis (IgM MGUS or SWM).

	IgM MGUS ^a^ (%) *n* = 64	SWM ^b^ (%) *n* = 107	*p*-Value
Serum M-protein (g/L) ≥12 g/L	12.1 51 (48)	11.7 37 (58)	0.6 0.1
Hemoglobin (g/dL)	13.3	13.4	0.8
Platelets (×10^9^/L)	255	245	0.4
β2-microglobulin (mg/dL) ≥4 mg/dL	2.9 6/60 (10)	2.4 5/96 (5)	0.1 0.25
Albumin (g/L) ≤35 g/L	42.3 3 (5)	42.5 4 (4)	0.7 0.7
Immunoparesis	4/63 (6)	10/104 (10)	0.4
Abnormal serum FLC ratio	10/32 (31)	32/60 (53)	0.04
*MYD88* L265P mutation	18/60 (30)	66/100 (66)	<0.001

^a^ IgM MGUS: IgM monoclonal gammopathy of undetermined significance; ^b^ SWM: smoldering Waldenström macroglobulinemia.

**Table 3:** Risk stratification model of progression to WM in patients with asymptomatic IgM monoclonal gammopathy as determined in the framework of death as a competing event.

**Adverse Variables**	**Subhazard Ratio (95% CI ^a^)**	***p*-Value**
Immunoparesis	10.2 (4.2–24.8)	<0.001
Lymphoplasmacytic infiltrate ≥20% in the bone marrow aspirate	6.0 (1.6–22.1)	0.007
**Risk Model**	**Subhazard Ratio (95% CI)**	***p*-Value**
Good prognosis group (no risk factors)	0.1 (0.02–0.5)	0.004

^a^ CI: Confidence interval.

## Data Availability

The data presented in this study are available in this article (and supplementary material).

## Supplementary Materials

The following are available online at https://www.mdpi.com/article/10.3390/cancers13092055/s1, Figure S1: Kaplan-Meier projected survival of 171 patients with asymptomatic IgM monoclonal gammopathy. Table S1: Association between immunoparesis and previously recognized risk factors for disease progression in IgM monoclonal gammopathy of undetermined significance.
