# Supplementary material for: Defining an Ultra-Low Risk Group in Asymptomatic IgM Monoclonal Gammopathy
Source: Cancers (Basel). 2021 Apr 23;13(9):2055. doi: 10.3390/cancers13092055 (PMC8122982; doi:10.3390/cancers13092055)
Supplement: Supplementary file 1 [file cancers-13-02055-s001.zip › cancers-1157569-supplementary.pdf]

Table S1. Association between immunoparesis and previously recognized risk factors for disease progression in asymptomatic IgM monoclonal gammopathy.

	**Immunoparesis (% or CI [a])**	**No immunoparesis (% or CI)**	***P*-value**
Male sex	5 (36%)	84 (55%)	0.2
Hemoglobin (g/dL)	13.0 (11.6–14.0)	13.4 (12.5–14.8)	0.2
Albumin (g/L)	40 (39–44)	43 (41–45)	0.06
Serum IgM (g/L)	18.0 (8.4–30.5)	4.9 (3.4–10.0)	<0.001
Serum M-Protein (g/L)	14.9 (11.1–24.0)	12.7 (9.0–14.6)	0.01
Abnormal FLC ratio [b]	4 (100%)	37 (43%)	0.025
Lymphoplasmacytic infiltration (%)	29 (10–54)	16 (11–23)	0.008
MYD88 mutation [c]	7 (53%)	75 (54%)	0.9

[a] CI: Confidence interval; [b] FLC: free light chain ratio (available in 92 patients); [c] Available in 160 patients.

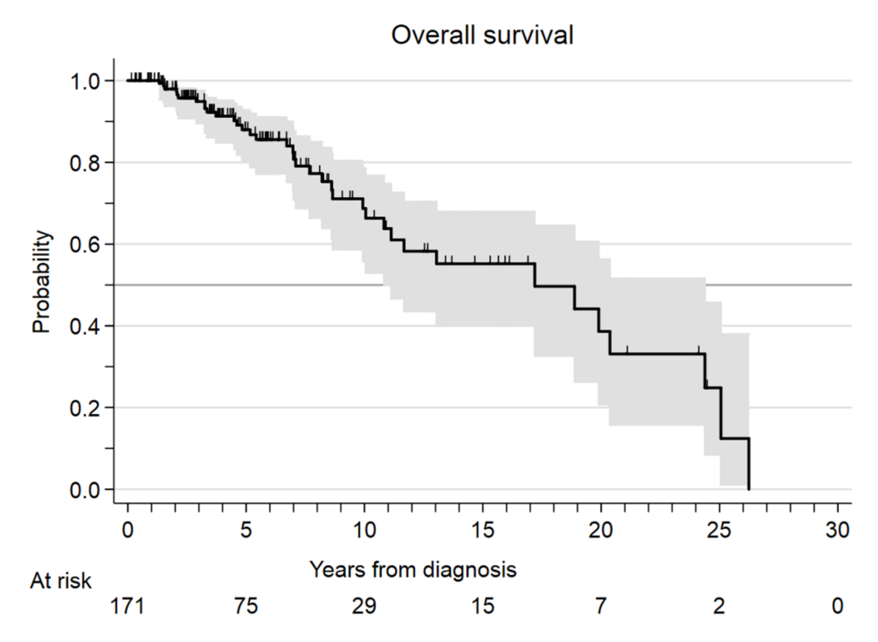

Figure S1. Overall survival of 171 patients with asymptomatic IgM monoclonal gammopathy.
